# Proteomics for the Identification of Biomarkers in Testicular Cancer–Review

**DOI:** 10.3389/fendo.2019.00462

**Published:** 2019-07-12

**Authors:** Domenico Milardi, Giuseppe Grande, Federica Vincenzoni, Francesco Pierconti, Alfredo Pontecorvi

**Affiliations:** ^1^International Scientific Institute “Paul VI”, Rome, Italy; ^2^Division of Endocrinology, Fondazione Policlinico'A. Gemelli' IRCCS, Rome, Italy; ^3^School of Medicine, Biochemistry and Clinical Biochemistry Institute, Catholic University of Rome, Rome, Italy; ^4^Department of Laboratory Diagnostic and Infectious Diseases, Fondazione Policlinico'A. Gemelli' IRCCS, Rome, Italy; ^5^Division of Anatomic Pathology and Histology, School of Medicine, Catholic University of Rome, Rome, Italy

**Keywords:** testis cancer, proteomics, biomarker, germ cell tumor, proteins

## Abstract

A large number of biomarkers have been proposed for the diagnosis of testicular cancer, representing putative molecular targets for anticancer treatments. However, no conclusive data have been provided. Proteomics represents a research field recently developed. It evaluates the large-scale analysis of the full protein components of a single cell, of a specific tissue, or of biological fluids. In the last decades, proteomics has been applied in clinical fields, thanks to modern technology and new bioinformatic tools, to identify novel molecular markers of diseases. The aim of this review is to argue the findings of recent studies in the discoveries of putative prognostic and diagnostic markers of testis cancer by proteomic techniques. We present here a panel of proteins identified by proteomics which might be used after validation for early detection and the prognostic evaluation of testicular tumors. In addition, the molecular mechanisms revealed by these proteomic studies might also guide the development of novel treatments in future.

## Introduction

Testicular germ cell tumors (TGCT) is the most frequent cancer occurring in young men. TGCTs are classified into two subgroups: non-seminoma and seminomas germ cell tumors. Seminomas rate for 50% of testicular cancer and non-seminoma germ cell tumors rate for 40% of testicular cancer. The remaining 10% of testicular cancer is associated with tumors and they usually include both seminoma and non-seminoma components (1). The difference in the diagnosis between seminoma and non-seminoma is fundamental for the objective of treatment and of prognosis.

TGCTs originate from transformed gonocytes or undifferentiated spermatogonia, but the pathogenesis of TGCT remains unexplored (2). To date, accessible markers for the diagnosis and follow-up aftercare include a-fetoprotein (AFP), ßHCG and LDH. However, AFP and ßhCG have high specificity (90%) but often relatively low sensitivity. For this reason tumor markers alone are not able to detect many recurrences, indeed in about 40% of men with disease recurrence the levels of these markers are usually “normal.” The LDH appears to have poor diagnostic performance (3). Therefore, the discovery of novel clinical biomarkers would clearly help the early detection and the monitor of the disease.

The diagnosis of “cancer” can be challenging. In addition to histopathological interpretation and immunohistochemical stains for confirming the precise cell of origin (4), more recently global gene expression profiling has been applied in order to facilitate the treatment decisions and prognosis. One key application for patients with the primary disease is precise prognosis, which helps to divide patients into different risk groups and select both treatment and monitoring strategies. Usually, the prognosis is based on clinical parameters such as age and tumor stage. Recently, considerable attempts have been made to incorporate molecular information in the staging process for accurate prognosis. Different tumors are classified in accordance to similar gene expression patterns. They represent a “molecular signature,” composed by several tens or hundreds of genes, in particular with regard to cell morphology or tissue characteristics. Global gene expression is most easily measured using cellular RNA; on the other hand, protein expression profiling provides a more dynamic view, offering additional informations on protein-protein interactions, post-trasductional modifications and finally on protein abundance (5).

Testicular cancer has been mainly studied at a genetic level. However, proteomics represents a promising technology that could allow novel insight into the disease at the molecular level to increase the understanding of their function. In the present molecular era proteomic is evaluated as a crucial point in personalized medicine to identificate specific target proteins for the pathophysiological state. Moreover, modern bioinformatic analysis offers information about the involvement of the proteins in the biological pathways of the tumor (6). Proteomics analysis of testicular tumor compared to normal testicular tissue may create a platform for enhanced understanding of differentially expressed proteins which might represent potential biomarkers for cancer. Protein expression profiling is a powerful tool in clinical practice, particularly in identifying cancer biomarkers to help the diagnosis and to choose a personalized treatment and monitoring of patients.

In the field of testicular tumors, particularly, the studies are few and outdated. Currently the main problem in the identification of protein markers is the small number of proteins which have been associated to TGCT.

## Proteomic Technologies Applied in TGCT: Features and Performances

Technological advances in proteomics have improved sensitivity and multiplexing ability of the method, as well as the possibility of identifying protein interactions. These advances can be of help to understand the molecular mechanisms involved in TGCT. The use of proteomics technologies offers an appealing approach to the identification and development of new tools to be used in clinical practice, identified by the simultaneous comparison of hundreds or thousands of proteins. The development and use of performant sample preparation techniques together with the increasing availability of proteomics technologies and recent technical advances in mass spectrometry (MS) enable identification and quantification of proteins involved in the diseases, although they are expressed at low abundance (7).

Up to now the most common proteomics technologies applied in the studies about TGCT include “gel-based” proteomics such as 2D-PAGE and 2D-electrophoresis associated with mass spectrometry (MS). SELDI-TOF and High-performance liquid chromatography (HPLC) associated with tandem mass spectrometry (MS/MS) moreover have been used in two studies. One additional proteomic study was performed in serum by SELDI-TOF.

### Separation Techniques

#### 2D Gel Electrophoresis

2D gel electrophoresis detaches proteins consistent with their isoelectric point. A second, independent separation step is then performed, dependent on mass (molecular weight), using sodium dodecyl sulfate polyacrylamide gel electrophoresis. This 2D-PAGE method can be performed to develop samples at high resolution and on a large scale. It might represent a primary screening method to form hypotheses and to guide further researches. 2D gel electrophoresis is the most frequently performed technique for proteomic analysis, although some limitations which are related to protein solubility (i.e., membrane proteins not easily solubilized), poor protein separation in the initial pH gradient (too basic or too acidic proteins) and low and high molecular weight (8, 9).

To better identify the separated proteins, proteases may be used to digest bands or spots obtained 2D-electrophoresis. The smaller fragments are then ionized and analyzed by mass spectrometry (MS).

#### High-Performance Liquid Chromatography (HPLC)

HPLC is used with the aim to obtain a complete protein recovery, including small basic and hydrophobic types (9). In fact, protein separation is based on some specific protein properties (hydrophobicity, surface charges, specific amino acid sequences) (10). Connecting HPLC with a mass spectrometer it is possible to obtain the rapid separation and the comprehensive identification of components of a complex protein mixture, with the aim to deeply analyze a proteome.

### Proteomic Analysis

#### Mass Spectrometry (MS)

MS-based techniques can be used to study complex protein mixtures previously fractionated by electrophoresis or HPLC. This technology provides precise mass values by the measurement of the mass-to-charge ratio (m/z) of the ions generated from the peptides and the proteins. In recent years a significant technical improvement of mass spectrometers has been observed. Modern mass spectrometers, such as time of flight (TOF), Fourier transform ion cyclotron resonance (FT- ICR) and Orbitrap detectors provide extremely accurate masses of analytes (11). Makarov invented the Orbitrap in 1999, as a mass analyzer that couples high resolution with high mass-accuracy, a significant m/z range and a high dynamic range (12, 13). The high mass-accuracy of the Orbitrap significantly contributes to the amount of acquired data and the number of analytic approaches that can be connected to MS (14).

#### SELDI TOF-MS

Surface-enhanced laser desorption/ionization time-of-flight mass spectrometry (SELDI TOF-MS) has been used widely in biomarker discovery because its sensitivity. It requires moreover only a small amount of protein for analysis. This method decreases the sample complexity by studying proteins with a specific chemical property that allows them to be adsorbed in a known surface (e.g., a charged, hydrophobic, or functionalized affinity surface). The non-bound protein population is removed by extensive washing. The adsorbed sample is ionized and analyzed (15).

## Proteomics of TGCT Tissue

Table 1 summarizes the proposed putative markers which have been identified in TGCT tissue.

**Table 1:** Proteins identified through proteomics as putative markers in TGTC tissue.

**Protein**	**Gene**	**Molecular function (Gene Ontology)**	**Expressed in TGTC**	**Biological function in TGTC**	**Reference**
Glutathione S-transferase (GSTs) M3 protein	GSTM3	Protein binding	Downregulated in seminoma	Polymorphism of GSTM3 predict higher risk of TGCT development	Zimmermann (16)
Gamma-tubulin complex component 6	GCP6	Microtubule binding	Identified in seminomas but not in the normal tubules	Role for microtubule nuclation at the centrosome. Excessive number of centrioles have been associated with chromosomal instability.	Leman (17)
Cyclin-dependent kinase 10	CDK10	Protein binding; kinase activity	Identified in seminomas but not in the normal tubules	Involved in cell cycle regulation	Leman (17)
StAR-related lipid transfer protein 7	STARD7	Lipid binding	Absent in seminomas	Involved in metabolism and signaling. It might play a role in mantaining the differentiated form of the normal cells.	Leman (17)
Mab-3 doublesex- and Related Transcription Factor 1	DMTR1	Molecular function	Over-expressed in mixed germ cell-sex cord stromal tumore and spermatocytic tumor	Controller of mitotic proliferation of germ cells	Liu (18)
Piwi-Like RNA Mediated Gene silencing 1	PIWIL1	Protein and mRNA binding	Specific protein of TGCT	Involved in RNA silencing during translational activity. It improves DNA methylation.	Liu (18)
Transmembrane (C-Terminal) Protease, Serine 12	TMPRSS12	Serine-type endopeptidase activity	Specific protein of TGTC	Previously reported in prostate and liver cancer. Unknown biological mechanism.	Liu (18)
p21-activated kinase 4	PAK4	Protein binding	Overexpressed in embryonal carcinoma	Involved in cell protections from apoptosis	Castillo (19)

For the first time in 2006 Zimmermann et al applied proteomic analysis to neoplastic germ cell tissue (16). This strategy has been applied to identify differences in the proteomes obtained by non-neoplastic and neoplastic tissues. Protein extracts have been analyzed by 2DPAGE combined with mass spectrometry. The comparative study permitted the identification and detection of quantitative differences of expressed proteins between seminoma and non-seminoma tissue.

Glutathione S-transferase (GSTs) M3 protein has been downregulated in seminoma tissues. GSTs detoxification enzymes partecipate in the pathogenesis of cancers. GSTM3 is a critical GSTs variant and previous evidences showed that GSTM3 polymorphism is associated with an increased risk to develope a cancer (20, 21). A lot of studies previously investigated the association of GSTM3 gene polymorphism with the risk to develop a lung cancer. Furthermore, a reduction in GST protein level was earlier reported in human TGCT (22), but the M3 isoform has not been previously identified in TGCT. Only proteomics permitted the identification of this specific isoform, as possibly associated with testis cancer. The M3 homodimer is a specific isoform specifically identified in the brain and in the testis (23). The GSTM3 gene is polymorphic and GSTM3 polymorphisms control the enzyme activity by the modulation of substrate binding (24). Distinct polymorphisms of GST-M3 enzyme are associated with a higher risk for TGCT formation (16). In particular, the study published in 2009, performed on a large population of patients who survived TGCT, reported that GSTP1 genotype influences the risk of developing a TGCT (25). M3 and P1 polymorphisms of GSTM3 represent promising markers for predicting the risk of TGTC formation.

In 2009 Leman *et al*. used a proteomics analysis to discover a pattern of proteins related with nuclear changes in seminoma cells (17). The changes in the form and in the size of the nucleus are trademarks of the cancer cell. Proteomics approach focused to mark a specific pattern of proteins related with the specific alterations in the nuclear structure of seminoma cells. Using high-resolution two dimensional gels, four nuclear matrix proteins have been identified in seminomas, but not in the normal tubules and three of these four proteins are part of gamma-tubulin complex component 6 (GCP6).

The γ-tubulin complex is a large multiprotein complex which plays an essential role in microtubule nucleation at the centrosome. C-tubulin ring complex (c-TuRC) is a key component of the centrosome which nucleates microtubules. GCP6 is localized in the pericentriolar material, a protein matrix composed by protein complexes involved in centrosome-associated functions (i.e., microtubule nucleation). Moreover, GCP6 is needed for centriole duplication and Polo-like kinase4 (Plk4)-induced centriole over-duplication. GCP6 interacts in fact with Plk4 and it is phosphorylated by the same Plk4. Therefore, controlling centriole numbers GCP6 preserves cells to have the correct number of centrosomes and cilia. Excessive number of centrioles lead to tumorigenesis in flies (26) and has been associated with chromosomal instability in humans (27).

Another specific protein for seminomas is Cyclin-dependent kinase 10 (CDK10). CDK10 is a Cdc2-related kinase and is a key element in the advance from the G2 to M phase transition of the cell cycle. Two isoforms of CDK10 have been documented: HCDK10-1 and HCDK10-2 (17). CDK10 is highly represented in colorectal cancer where it takes part in the suppression of apoptosis and in the stimulation of tumor growth *in vitro* and *in vivo*. The modulation of CDK10 expression in colorectal cancer indicates that CDK10 is involved in cell growth and it is associated with a reduction in chemosensitivity. Finally it inhibits apoptosis through the upregulation of the expression of Bcl-2 (28). Both the CDK10 isoforms are expressed in the nuclear matrix of the seminomas, supporting the role of CDK10 in the cell cycle regulation that may induce testicular cancer.

In normal testis moreover seven specific nuclear matrix proteins have been detected, which are absent in seminoma tissue. Some of these proteins are testis specific: Y177 encoded-like protein 4, cytokeratins, glutamine synthetase, and StAR-related lipid transfer protein 7 (StarD7).

StarD7 has been reported for the first time as related with nuclear matrix and it is part of the family of StAR1-related lipid transfer (START) proteins. These proteins are involved in lipid transport, metabolism and signaling (17). Overexpressed StARD7 gene has been specifically linked with colorectal cancer. StarD7 protein upregulation has been documented in differentiating cytotrophoblast suggesting that StarD7 might have a function in throphoblast differentiation through phospholipids uptake and transport (29). The loss of StarD7 protein in JEG-3 cells alters ABCG2 multidrug transporter level, cell migration, cell proliferation, and differentiation marker expression. StarD7, expressed in normal testis but not in seminoma tissue, might have a role in maintaining the differentiated form of the normal cells. The loss of StarD7 might induce cancer development.

The testicular microenvironment is a unique environment. Spermatogenesis and tumorigenesis at testicular level in humans are two biological processes which have got numerous similarities between them. It is very important to know the common mechanisms between the two biological processes. In 2013 Liu *et al*. performed an extensive study for tumor marker identification by proteomic of testicular tissues. Using 2D-high performance liquid chromatography (HPLC)–MS/MS (LTQ Orbitrap Velos hybrid mass spectrometer) the Authors identified 7,346 proteins in testis tissue with normal spermatogenesis (18).

The protein data were confirmed by immunohistochemistry and by comparison with previously published data from the Human Testis Proteome Database. These date have been confirmed by using of a GWAS study, using associated SNPs in case of differential expression of these proteins. Among these testicular proteins, six novel cancer/testis gene transmembrane protease have been characterized: serine 12 (TMPRSS12), tubulin polymerisation promoting protein family member 2 (TPPP2), protease serine 55 (PRSS55), double-sex and mab-3 related transcription factor 1 (DMRT1), piwi-like RNA-mediated gene silencing 1 (PIWIL1), and hemogen (HEMGN). The last four proteins have been proposed to exert a central role in spermatogenesis and cancer development, although they still haven't known specific functions in testis (18).

The Mab-3 doublesex- and Related Transcription Factor 1 (DMTR1) has been associated to prostate cancer. It is a controller of mitotic proliferation in germ cells, thanks to a specific zinc finger structural motif which is associated with cell cancer proliferation. A recent study confirmed these results in the human testis; nuclear expression of DMRT1 was reported in spermatogonia, but not in primary spermatocytes that have entered meiosis 1 or in more mature germ cells. DMRT1 expression was confirmed in the germ cells of testicular mixed germ cell–sex cord stromal tumor (MGC-SCST) and spermatocytic tumor but not in those of seminoma. For this reason the germ cells of MGC-SCST are related to spermatogonia, which espress DMRT1. The strong expression of DMRT1, together with the absence of TCLF5 in the germ cells of both MGC-SCST and spermatocytic tumor, suggests a premeiotic origin for both tumors (30).

The second protein is Piwi-Like RNA Mediated Gene silencing 1 (PIWIL1) which, together with the allelic variant rs10773777, was also detected in prostate cancer cases. PIWIL1 was also detected by proteomic approach as a specific protein of TGCT. This protein might play a pivotal function in RNA silencing during the modulation of tranlational activity. Previous studies reported that PIWIL1 plays an important role in cancer development, improving DNA methylation. Several cancer-germline genes have been defined to stimulate PIWIL1 as a part of oncogenic pathways involved in cell proliferation (31). More recently PIWIL1 expression was demonstrated in spermatocytes and spermatids. Up to 70% of TGCT samples express PIWIL1, which is not normally expressed in premeiotic germ cells. These evidences support that in many germ tumors is present an aberrant expression of PIWIL1. The enhanced expressions of piwil2 was found in seminomas and has not been reported in testicular non-seminomas tumors (31).

The third protein is Transmembrane (C-Terminal) Protease, Serine 12 (TMPRSS12). It was previously related with colorectal cancer for the variant rs11169552 (32). This protein has been demonstrated to be expressed in spermatids and spermatocytes (33). The Tubulin Polymerization-Promoting Protein Family Member 2 (TPPP2) and its variant rs1952524 was linked to liver cancer (34). Another protein is the Protease Serine 55 (PRSS55) along with the variant rs4404875, which has been mainly identified in Leydig and Sertoli cells and it is is associated with prostate and ovarian cancer (35). Finally Hemogein (HEMGN), which regulates proliferation and differentiation in hematopoietic cells, has been associated with thyroid cancer (36). Among the 300 proteins expressed in human testis, only 22.7% are TGCT-related proteins, of which only 65 proteins have been evaluated, indicating that other candidate proteins exist, although not still studied, The functional analysis of only 65 out 300 proteins might depend to still low sensitive methods in the field of proteomics. The six proteins previously cited might represent moreover important targets for personalized therapy in this kind of neoplasy (18).

In a very recent study, Castillo *et al*. identified 174 phosphorylated kinases in human testis by metal oxide affinity chromatography using TiO2 combined with LC-MS/MS. Protein phosphorylation is involved in the modulation of cell cycle, cell growth, cell differentiation and cell death. Two kinases have been studied in the testis phosphoproteome as candidates for further studies by immunodetection procedures. Immunodetection has been specifically used to study the potential function of cyclin dependent kinase 12 (CDK12) and p21-activated kinase 4 (PAK4) in testicular tissue. The in silico protein-protein interactions have been studied, and a functional analysis in a human embryonal carcinoma cell line has been performed. PAK4 is localized in human spermatogonia. Its function in preventing the activation of caspase is well-known. In embryonal carcinoma it has been observed that PAK4 protects cells from apoptosis. PAK4 inhibitors might represent an interesting pharmacological target for novel drugs modulating behavior of testicular cancer (19).

## Proteomics of TCGC in Serum

Protein markers that distinguish cancer patients by healthy controls can be moreover searched in serum.

A single serum proteomic study regarding testis cancer was carried out. In 2010, Strenziok et al utilizing Surface-enhanced laser desorption ionization time-of-Xight mass spectrometry (SELDI-TOF MS) identified the protein profiles of TGCT patients that are different in a highly significant degree from normal subjects (37). CM10 ProteinChip® array identified 138 peaks in a mass range of 3,800–10,000 Da that might repredent a “molecular fingerprint” to differentiate tumor serum sample from non-tumor serum samples. The spectra of proteins have been investigated by the proteomic platform “proteomic.net.” Five peaks have been verified by CM10 ProteinChip® (6.48, 6.84, 8.15, 8.17, 8.92 kDa). There was no single peak that could discriminate the group of seminoma vs. control subjects, so a cluster classifications has been performed.

For statistical analysis, an artificial intelligence learning algorithm used three different bioinformatics methods to develop the training set for the decision trees, support vector machines, and neural networks and to differentiate between the two groups. Decision tree analyses developed the most powerful classifier with 89.4% specificity and 91.5% sensitivity (CM10 ProteinChip®, 95% confidence interval of 82.6–95.5%).

In this study the authors after the first step in investigation separating cancer from healthy controls with the definition of protein profiles of TGCT patients that differ in a highly significant degree from normal controls; the protein identification of peak masses was not necessary to differentiate cancer patients from healthy subjects.

Validation of these results may permit proteomic profiling to become a useful tool especially for aftercare follow up.

## Conclusions

In the past 20 years molecular biomarker identification achieved importance in the field of personalized medicine, aimed to identify a cancer in the early stages and to develop novel therapeutical approaches. According to these premises, the discovery of novel specific markers would help the management of patients with testicular cancer.

Proteomics has proven to represent a promising platform for identifying biomarkers linked to testicular cancer.

The conventional tumor markers AFP, hCG, and LDH have demonstrated value in the clinical management of testicular malignant TGCT. However, their limitations in sensitivity and specificity prevent more universal application, especially in patients with seminoma. Tissue and serum biomarkers show exciting promises in the identification of markers for the diagnostics of TGCT. Furthermore, no proteomic studies have been permorfed aimed to detect markers of TGCT in semen, although it represents a promising source of putative biomarkers in different clinical situations (38–40).

Proteomic identification of TGCT-related proteins will allow to validate candidate markers for early detection and the prognostic evaluation of testicular tumors. In addition, the molecular mechanisms revealed by these proteomic studies might also guide the development of novel treatments in future.

## Author Contributions

DM and GG: conceptualization and writing—original draft preparation. DM and FP: literature analysis. FV and AP: writing—review and editing. AP: supervision.

### Conflict of Interest Statement

The authors declare that the research was conducted in the absence of any commercial or financial relationships that could be construed as a potential conflict of interest.
